# Tribological Properties of Flame-Sprayed Fe-Based Composite Coatings Synergistically Enhanced by NiCr-Cr_3_C_2_ and Ni-MoS_2_

**DOI:** 10.3390/ma19204290

**Published:** 2026-10-12

**Authors:** Xiqiang Ma, Hongyang Wei, Nan Guo, Jinpeng Li, Dongliang Jin

**Affiliations:** 1 School of Mechatronics Engineering, Henan University of Science and Technology, Luoyang 471000, China; maxiqiang@haust.edu.cn (X.M.); 15936029374@163.com (H.W.); guonan1860@163.com (N.G.); 2 Xinchang Technology Achievement Transformation Center, Henan University of Science and Technology, Xinchang 312500, China; ghost2233@163.com; 3 Zhejiang Sling Intelligent Drive Group Co., Ltd., Xinchang 312500, China

**Keywords:** flame spraying, Fe60-based composite coating, NiCr-Cr_3_C_2_ cermet, Ni-MoS_2_ core–shell powder, tribological properties

## Abstract

**Highlights:**

**What are the main findings?**

• The hard skeleton and lubricating products synergistically achieve a balance between friction reduction and wear resistance.
• Among the nine investigated compositions, C6 (15 wt.% Ni-MoS_2_ + 40 wt.% NiCr-Cr_3_C_2_) showed the best measured tribological performance under the present test conditions.
• C6 exhibited a friction coefficient of 0.50 and a wear rate of 0.59 × 10^−4^ mm^3^/(N·m).

**What are the implications of the main findings?**

• The tribological response depends on an appropriate balance among the hard phase, Mo/S-containing components, and the Fe60 matrix.
• The findings provide a compositional reference for Fe-based composite coatings under the investigated dry-sliding conditions.
• This offers new insights into the application of MoS_2_-based composite powders for thermal spraying.

**Abstract:**

Moving parts in mechanical equipment are susceptible to wear under sliding friction, which can reduce service life and operational reliability. Although Fe-based alloy coatings provide high hardness and relatively low cost, their friction-reduction capability remains limited, while direct incorporation of solid lubricants may result in thermal decomposition and phase transformation during high-temperature spraying. In this study, a Fe-based composite coating was designed using Fe60 as the matrix, NiCr-Cr_3_C_2_ as a wear-resistant reinforcing phase, and Ni-coated MoS_2_ as a source of Mo/S-containing lubricating components. The coatings were fabricated by flame spraying, and the effects of composition on their microstructure, phase constitution, hardness, and tribological properties were systematically investigated. The results show that MoS_2_ undergoes substantial thermal transformation during spraying, accompanied by the formation of Mo- and S-containing phases. The tribological behavior was further evaluated by combining friction and wear measurements with worn-surface SEM/EDS, XRD, and two-dimensional cross-sectional wear-track profiles. Among the nine investigated formulations, the coating containing 15 wt.% Ni-MoS_2_ and 40 wt.% NiCr-Cr_3_C_2_ (C6) exhibited the lowest measured friction coefficient of 0.50 and a wear rate of (0.59 × 10^−4^) mm^3^/(N·m), together with the highest measured microhardness. The relatively shallow wear-track profile of C6, together with the retention of Cr/Ni-rich hard phases and the presence of Mo/S-containing species on the worn surface, supports an evidence-based interpretation in which the Cr_3_C_2_-containing phase provides load-bearing and wear resistance, the Mo/S-containing products may contribute to reducing interfacial shear resistance, and the Fe60 matrix provides continuous support and accommodates local deformation. These complementary effects contribute to the composition-dependent balance between friction reduction and wear resistance. The present findings provide a compositional reference for designing thermally sprayed Fe-based composite coatings with combined wear resistance and friction-reduction capability under the investigated dry-sliding conditions.

## 1. Introduction

As is widely recognized, numerous kinematic pairs in mechanical equipment remain in sliding contact throughout service, and their tribological performance directly governs system efficiency, reliability, and service life. Extending their wear life has become a research focus in the field of surface engineering [1]. Currently, surface strengthening via surface coating technologies is an effective approach [2]. Among these, thermal spraying is widely used for component surface protection [3] and is regarded as one of the most promising surface engineering technologies because it can heat and melt powder materials and deposit them onto metal substrate surfaces at high velocity [4], forming protective coatings with excellent tribological performance [5].

Fe60 Fe-based self-fluxing alloy coatings are widely used for wear-resistant reinforcement and repair of components because of their low cost, good metallurgical bonding with steel substrates, and high as-deposited hardness [6]. However, their high hardness mainly relies on a large amount of brittle carboboride hard phases, resulting in inherently insufficient toughness [7]; under repeated sliding contact stresses, stress concentration readily occurs around the hard phases and initiates microcracks [8], severely degrading the long-term service reliability of the coatings. Meanwhile, the coatings themselves exhibit a relatively high dry friction coefficient and lack self-lubricating components [9]; under boundary lubrication or oil-starved sliding conditions, adhesion and scuffing tend to occur at the friction interface, leading to premature failure of the friction pair [10]. Therefore, how to improve the toughness and friction-reducing performance of Fe60 coatings while maintaining their high hardness has become a key challenge in the compositional design of Fe-based coatings for sliding friction conditions.

MoS_2_ is a typical layered transition metal dichalcogenide, whose crystal structure consists of S-Mo-S sandwich layers with strong covalent bonding within each layer and weak van der Waals interactions between adjacent layers. This unique structure endows MoS_2_ with low interlayer shear strength and favorable friction-reduction characteristics. However, during flame spraying, MoS_2_ may not be fully retained in its original crystalline form and may undergo thermal decomposition, desulfurization, oxidation, or reactions with matrix elements. The resulting Mo/S-containing transformation products may subsequently participate in the tribological process and contribute to reducing interfacial shear resistance during sliding. MoS_2_ has low thermal stability, and its direct addition can degrade the structural integrity of the coating [11]. Ni-coated MoS_2_ with a core–shell structure consists of a layered MoS_2_ core fully encapsulated by a metallic Ni shell [12]. This structural design improves the interfacial compatibility of MoS_2_ [13]: although the Ni shell cannot prevent the thermal decomposition of MoS_2_, it can effectively confine the decomposition products within localized regions [14], suppress their excessive escape and splashing, and promote their wetting and dispersion within the Fe-based matrix. Meanwhile, the Ni shell converts the ceramic-like surface of MoS_2_ into a metallic surface that is metallurgically compatible with Fe60, thereby alleviating the critical drawbacks of poor wettability with Fe-based alloys and the tendency to act as crack initiation sites [15], and enabling firm bonding and uniform dispersion of the lubricating phase within the metallic matrix.

Commonly used wear-resistant cermets mainly include WC-Co, WC-CoCr, and Cr_3_C_2_-NiCr systems. Among them, WC-based coatings exhibit high hardness and excellent wear resistance, but the Co-based binder phase is prone to softening and failure under high-temperature oxidizing environments, limiting their application in complex service conditions. Although TiC-based cermets possess high hardness, their insufficient toughness makes it difficult to meet the demands of impact wear conditions. In contrast, Cr_3_C_2_-NiCr composite powders combine the abrasive wear resistance of the Cr_3_C_2_ hard phase with the toughness and oxidation resistance of the NiCr binder phase; the microstructure of NiCr-Cr_3_C_2_ cermet powders consists of high-hardness Cr_3_C_2_ ceramic particles uniformly dispersed in a tough NiCr alloy matrix [16]. The Cr_3_C_2_ particles impart excellent abrasive wear resistance by effectively resisting micro-cutting and plowing from hard abrasive particles [17]; the NiCr binder phase relieves stress concentration and blunts cracks through local plastic deformation, significantly enhancing impact spalling resistance and toughness [18]. Moreover, the good wettability and metallurgical compatibility between NiCr and Fe-based, Ni-based, and other metallic matrices ensure robust interfacial bonding and prevent premature spalling of the hard phase [19].

However, a single strengthening mechanism cannot satisfy the comprehensive demands for long-term service of components under complex sliding friction and wear conditions. For Fe60 Fe-based self-fluxing alloy coatings, increasing the hard phase content can enhance surface hardness and abrasive wear resistance; however, excessive brittle phases reduce the overall toughness of the coating, causing stress concentration at the interfaces between hard phases and the metallic matrix, and under cyclic friction loads, inducing crack initiation, propagation, and particle spalling. On the other hand, introducing solid lubricant phases can effectively reduce shear resistance at the sliding interface, but because the lubricant phases themselves have limited load-bearing capacity, excessive addition tends to weaken the continuous load-bearing skeleton of the coating, leading to localized softening and reduced interfacial stability. Therefore, how to achieve a rational match between load-bearing reinforcing phases and lubricating components while improving the wear resistance of the coating, and to balance hardness, toughness, and tribological stability, is a critical issue that urgently needs to be addressed in the design of Fe-based wear-resistant self-lubricating coatings.

To address these issues, this study proposes a multi-phase synergistic composite coating system consisting of an Fe60 matrix, a Cr_3_C_2_-NiCr hard reinforcing phase, and an Ni-MoS_2_ self-lubricating component. In this system, the Fe60 matrix serves as the continuous phase, providing plastic support and load-transfer capability to the coating. The Cr_3_C_2_-NiCr cermet reinforcing phase exploits the high hardness of Cr_3_C_2_ to enhance the coating’s resistance to micro-cutting and plastic deformation during friction and wear, while the NiCr binder phase alleviates stress concentration between hard particles and the matrix, thereby improving interfacial bonding strength and spalling resistance. The Ni-coated MoS_2_ lubricating component improves the interfacial compatibility between MoS_2_ and the metallic matrix, enhancing its stability during spraying and service; during sliding, Mo/S-containing transformation products derived from the Ni-MoS_2_ component may contribute to reducing shear resistance at the friction interface. The synergistic control of the hard reinforcing phase and the solid lubricant phase is expected to balance wear resistance and friction reduction, providing a compositional reference for Fe-based composite coatings under the investigated dry-sliding conditions.

In this study, Fe60-based composite coatings containing different proportions of Ni-MoS_2_ and NiCr-Cr_3_C_2_ were prepared by flame spraying. The objective was to systematically determine how the relative contents of these two functional components affect the microstructure, phase constitution, microhardness, friction behavior, and wear performance of the coatings. The combined results of SEM/EDS, XRD, microhardness, friction and wear measurements, and wear-track profilometry were further used to interpret the complementary roles of the hard phase, Mo/S-containing products, and Fe60 matrix during sliding.

## 2. Materials and Methods

### 2.1. Materials Preparation

A commercially available Fe60 Fe-based self-fluxing alloy powder (Jincan Metal, China) with a particle size range of 45–106 μm was used as the coating matrix powder, and its chemical composition is listed in Table 1. This powder is mainly composed of Fe, Cr, Ni, B, Si, and C, with Cr and Ni contents of 16–18 wt.% and 9.0–12 wt.%, respectively, and B, Si, and C contents of 3.8–4.2 wt.%, 1.0–2.0 wt.%, and 0.8–1.2 wt.%, respectively, with Fe as the balance. A commercially available NiCr-Cr_3_C_2_ cermet composite powder (Bocheng New Materials, China) with an average particle size of approximately 5 μm was introduced as the hard reinforcing phase, and its chemical composition is shown in Table 2. This powder mainly consists of a Cr_3_C_2_ hard phase and an NiCr metallic binder phase, with Cr, Ni, and C contents of 68.84 wt.%, 21.36 wt.%, and 9.69 wt.%, respectively, and Fe and O impurity contents of 0.06 wt.% and 0.05 wt.%, respectively. A commercially available Ni-MoS_2_ composite powder (Hebei Chaoyue Metal Alloy Materials Co., Ltd., China) with a particle size range of 30–100 μm was selected as the solid lubricant component, and its chemical composition is presented in Table 3. This powder exhibits a typical Ni-coated MoS_2_ composite structure, with an Ni content of 72–80 wt.% and MoS_2_ as the balance.

To investigate the effects of different Ni-MoS_2_ and NiCr-Cr_3_C_2_ contents on the microstructure and tribological properties of Fe60-based composite coatings, nine groups of coating specimens with different compositional ratios were designed, as listed in Table 4. All specimens used Fe60 Fe-based self-fluxing alloy powder as the matrix phase, and composite coating systems with different compositional gradients were constructed by adjusting the addition ratios of the Ni-MoS_2_ solid lubricant component and the NiCr-Cr_3_C_2_ cermet reinforcing phase. Here, C1–C9 represent coating samples with different proportions.

As shown in Table 4, the Fe60 content varied within the range of 40–70 wt.%, while the Ni-MoS_2_ content was set at three levels of 10 wt.%, 15 wt.%, and 20 wt.%, and the NiCr-Cr_3_C_2_ content was correspondingly adjusted to 20 wt.%, 30 wt.%, and 40 wt.%. Specifically, for specimens C1–C3, the Ni-MoS_2_ content was maintained at 10 wt.%, and the Fe60 proportion was reduced by gradually increasing the NiCr-Cr_3_C_2_ content from 20 wt.% to 40 wt.%; for specimens C4–C6, the Ni-MoS_2_ content was increased to 15 wt.%, and the NiCr-Cr_3_C_2_ content was varied to explore the role of the hard reinforcing phase at a moderate lubricant content; for specimens C7-C9, the Ni-MoS_2_ content was further increased to 20 wt.% to analyze the influence of a higher lubricant content on the coating structure and tribological properties.

This compositional design achieves systematic control over the contents of the NiCr-Cr_3_C_2_ hard reinforcing phase and the Ni-MoS_2_ solid lubricant phase, thereby enabling the synergistic mechanisms between the hard load-bearing phase and the lubricating functional phase, as well as the effects of different addition ratios on the hardness, microstructure, and tribological behavior of Fe60-based composite coatings, to be investigated.

To ensure uniform powder feeding and homogeneous coating composition during spraying, the Fe60, NiCr-Cr_3_C_2_, and Ni-MoS_2_ powders were first mechanically mixed according to the designed ratios for 2 h. Thorough mixing reduces agglomeration and compositional segregation among the different powders, improves the homogeneity of the composite powder, and facilitates the formation of a coating with a uniform microstructure. The mixed composite powders were dried in a DZF-type vacuum drying oven (Beijing Kewei Yongxing Instrument Co., Ltd., China) at 150 ± 3 °C for 3 h. The temperature of 150 °C was selected because it is sufficiently high to remove physically adsorbed moisture and volatile impurities from the powder surfaces, but well below the temperatures at which MoS_2_ oxidizes (~315 °C in air) or decomposes (~780 °C in an inert atmosphere), and below the temperature at which Fe60 or NiCr-Cr_3_C_2_ powders undergo significant oxidation or sintering. Vacuum drying of metal powders at 150–200 °C is a standard pretreatment to eliminate adsorbed moisture without causing secondary oxidation. A holding time of 3 h ensures sufficient heat transfer and moisture removal from the relatively coarse Fe60 powder (45–106 μm) and from porous agglomerated particles. Drying at 150 °C for 2–3 h has been reported for Fe-based alloy powders prior to thermal spraying or laser cladding [20]. Residual moisture can cause powder agglomeration, unstable feeding, and increased coating porosity due to steam formation during spraying. The vacuum environment (<133 Pa) further reduces the oxygen partial pressure, preventing surface oxidation during drying.

To further enhance the mechanical interlocking between the coating and the substrate and thereby improve coating adhesion, the substrate surface was grit-blasted with 160-mesh white alumina abrasive prior to spraying. Grit blasting increases the surface roughness of the substrate, providing more mechanical interlocking sites for molten powder particles and thus improving coating deposition stability. After grit blasting, the substrate was cleaned again and dried to prevent residual abrasive and contaminants from affecting the coating microstructure and properties.

The flame-spraying parameters strongly affect the melting behavior of the feedstock particles and, consequently, the density and microstructure of the deposited coating. The main parameters include flame temperature, fuel-oxygen ratio, spray distance, particle velocity, and powder characteristics. An appropriate flame temperature increases the particle temperature and molten fraction, promoting droplet spreading and filling of surface asperities and interlamellar gaps, thereby reducing porosity and increasing coating density. Particle velocity further influences the impact kinetic energy and flattening behavior of the molten droplets; a sufficiently high velocity promotes spreading and densification of the deposited layer. However, excessive temperature or velocity may increase oxidation, splashing, or residual stress. The spray distance determines the residence time of particles in the flame and their thermal and kinetic losses during flight; an excessively long distance may result in insufficiently molten particles and reduced spreading, whereas an excessively short distance may impose excessive thermal input on the substrate. In addition, particle size strongly affects heating and melting because finer particles generally respond more rapidly to the flame. Therefore, the flame-spraying parameters should be appropriately balanced to obtain a suitable combination of particle temperature, molten fraction, and velocity, thereby promoting effective splat formation and reducing coating porosity. These relationships between spray parameters, particle state, splat formation, and coating porosity have been widely reported in thermal spray studies [21,22].

Subsequently, the mixed powders were deposited onto a 65Mn steel substrate by flame spraying. The 65Mn steel substrate was supplied by HBIS Company Limited, China, and its main chemical composition is 0.62–0.70 wt.% C, 0.17–0.37 wt.% Si, and 0.90–1.20 wt.% Mn, with Fe as the balance (Table 5). During spraying, the gun moved along a fixed scanning path from left to right and from top to bottom to ensure uniform coating thickness and microstructure. The flame spraying parameters are listed in Table 6. Propane (purity > 99.5%) and oxygen (purity > 99.5%) were supplied by Luoyang Nengliang Industry and Trade Co., Ltd., China. The propane flow rate was 19 L/min, the oxygen flow rate was 12 L/min, the gun traverse speed was 350 mm/s, the powder feed rate was 50 g/min, and the spray distance was 130 mm. By controlling these parameters, the powder particles were fully heated and melted in the flame, and upon high-velocity impact with the substrate, formed a continuous and dense lamellar structure. The resulting composite coatings had a thickness of approximately 0.2 mm.

### 2.2. Characterization

Multiple characterization techniques were employed to systematically analyze the microstructure, phase composition, elemental distribution, and mechanical properties of the prepared coatings. The surface and cross-sectional morphologies of the nine composite coatings were observed using scanning electron microscopy (SEM, ZEISS Sigma 300, Germany) to analyze the lamellar stacking structure, distribution of reinforcing phases, particle bonding state, and microstructural features such as pores and defects within the coatings. In addition, energy dispersive spectroscopy (EDS) attached to the SEM was used for point analysis and elemental mapping of the coating cross-sections and worn regions to examine the distribution characteristics of key elements such as Fe, Cr, Ni, Mo, and S, providing a basis for analyzing the elemental migration behavior of the Ni-MoS_2_ component during spraying and friction.

In the experiment, for each coating component (C1–C9), a random cross-sectional area was selected for analysis. A total of nine sets of area distribution measurement data were obtained. These element distribution maps represent the results of a single selected area, rather than the average of multiple measurements, and no point analysis results were used to generate these maps.

The phase composition of the coatings was analyzed using an X-ray diffractometer (XRD, Bruker D8 Advance, Germany) to identify the main crystalline phases formed during flame spraying and possible phase transformations. The XRD measurements were performed using Cu Kα radiation (*λ* = 0.15406 nm) over a 2*θ* range of 10–90° at a scanning rate of 5°/min.

Additionally, the cross-sectional microstructure of the coatings was observed using an OLYMPUS-BX53M optical microscope to analyze coating thickness, lamellar structure, and interfacial bonding state. The microhardness of the coatings was measured using an HVT-1000 microhardness tester (Laizhou Huayin Testing Instrument Co., Ltd., China) to evaluate the effects of different NiCr-Cr_3_C_2_ and Ni-MoS_2_ addition ratios on the mechanical properties of the coatings.

The thickness of the flame-sprayed coatings was approximately 0.2 mm (200 μm). To minimize the influence of the 65Mn substrate on the measured coating hardness, the relative indentation depth was controlled according to the commonly adopted criterion (*h*/*t* < 0.10), where (*h*) is the indentation depth and (*t*) is the coating thickness [23]. Accordingly, the maximum allowable indentation depth was approximately 20 μm. For the Vickers indenter, the indentation depth was estimated from the average indentation diagonal (*d*) using (*h* = *d*/(2tan68°)); thus, an indentation depth of 20 μm corresponds to an average indentation diagonal of approximately 99 μm. The indentation dimensions were examined after testing to verify that (*h*/*t* < 0.10). At least five indentations were made at different locations on each coating, and the average value was used as the representative microhardness.

### 2.3. Friction and Wear Test

To ensure the accuracy and reproducibility of the wear-test results, the coated specimens were ultrasonically cleaned with anhydrous ethanol before testing to remove surface contaminants and loosely attached debris, followed by thorough drying. Dry reciprocating sliding tests were conducted using an MF-5000 friction and wear tester to investigate the tribological behavior of the composite coatings with different compositions under unlubricated conditions. A Si_3_N_4_ ceramic ball with a diameter of 3.6 mm was used as the counterpart because of its high hardness, good wear resistance, and chemical stability, which help reduce the influence of counterbody wear on the measured coating wear. Si_3_N_4_ balls have also been widely used as counterbodies in dry reciprocating tribological tests of coatings and hard materials.

The reciprocating ball-on-flat configuration was selected with reference to ASTM G133-22 [24], which covers sliding wear tests of metals, ceramics, and other wear-resistant materials and recommends that the normal load and sliding speed be selected according to the intended application severity and the purpose of the test. Accordingly, a normal load of 40 N was selected to generate sufficient contact stress and measurable wear while avoiding excessively severe damage to the coating. Previous dry reciprocating wear tests using Si_3_N_4_ ceramic balls have employed comparable load levels for evaluating coating wear behavior [25]. A reciprocating frequency of 5 Hz and a stroke length of 6 mm were selected to provide a stable and reproducible sliding condition. Under these conditions, the corresponding mean sliding speed is approximately 0.06 m s^−1^. A test duration of 30 min corresponds to 9000 reciprocating cycles and a total sliding distance of approximately 108 m, which is sufficient to produce measurable wear while avoiding excessively prolonged testing. Similar 6 mm stroke and 30 min reciprocating wear conditions have also been reported in tribological testing using an MFT-5000-type tester [26].

Therefore, the final test parameters were fixed at a normal load of 40 N, a reciprocating frequency of 5 Hz, a stroke length of 6 mm, and a test duration of 30 min. The same conditions were applied to all coating compositions to ensure consistent test severity and enable a direct comparison of their friction and wear behavior.

For quantitative comparison, the average coefficient of friction (COF) was calculated as the arithmetic mean of the COF data recorded during the relatively steady sliding period from approximately 300 to 1800 s, with the initial running-in period (0–300 s) excluded [27].

After the wear tests, the wear track morphology was measured using a precision profilometer, and the wear volume was calculated. Based on the Archard wear model, the wear resistance of the coatings was evaluated using the volumetric wear rate [28], calculated as follows:

(1) $$W=\frac{V}{F\cdot L}$$

where *W* is the volumetric wear rate (mm^3^/(N·m)), *V* is the wear volume (mm^3^), *F* is the normal load (N), and *L* is the total sliding distance (m). This parameter eliminates the influence of differences in load and sliding distance on wear results, enabling a more accurate evaluation of the wear resistance of different composite coatings.

After the wear tests, the wear track morphology was observed by SEM, and the elemental composition and distribution in the worn regions were analyzed by EDS to further elucidate the wear mechanisms of the composite coatings.

## 3. Results

### 3.1. Microstructure of Powders and Coatings

**Figure 1:**
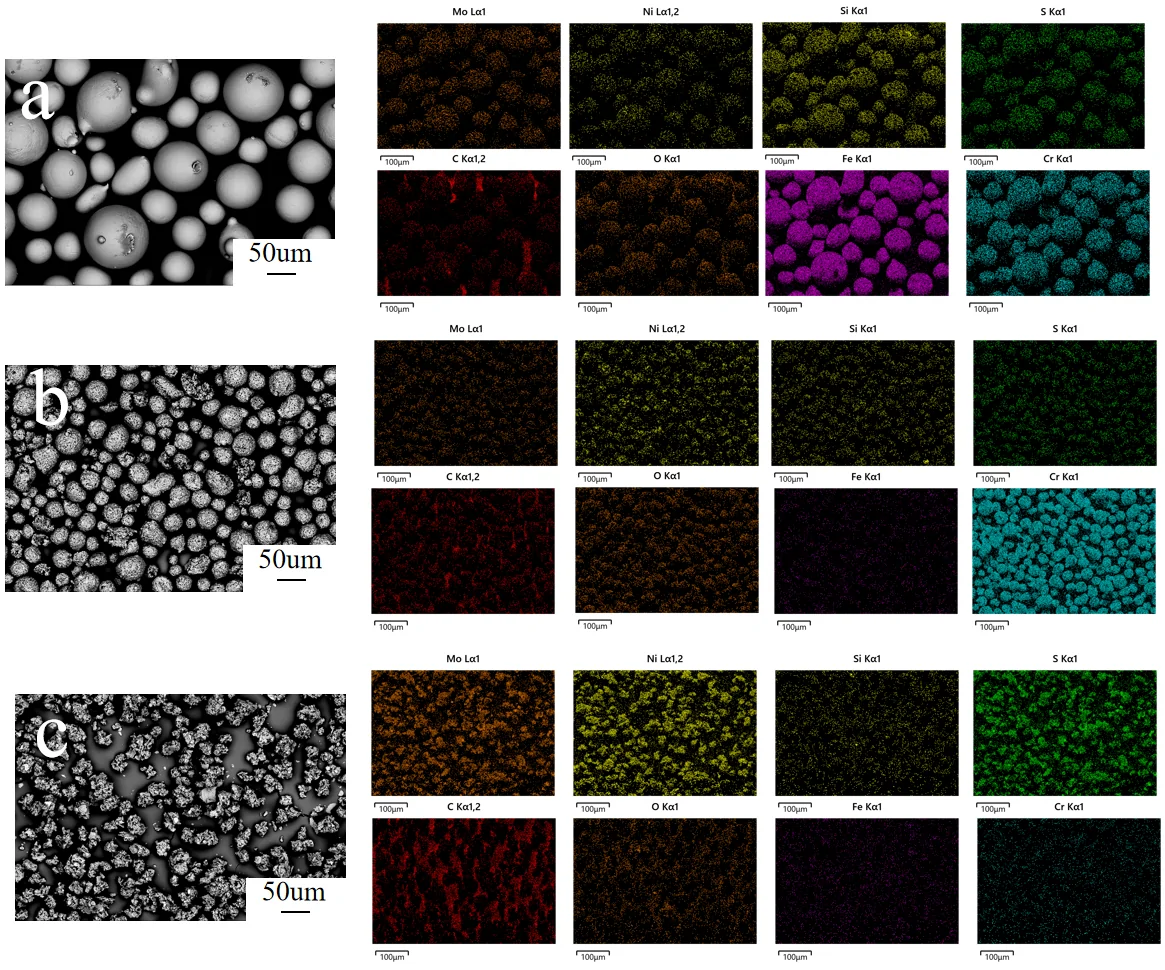
SEM micrographs of the different powders and EDS elemental mappings of randomly selected regions: (**a**) Fe60 powder, (**b**) NiCr-Cr_3_C_2_ powder, and (**c**) Ni-MoS_2_ powder.

Figure 1a shows that the Fe60 powder exhibits a nearly spherical or irregular morphology and consists of a solid alloy containing Fe, Cr, B, and Si, with a particle size of approximately 50 μm and good flowability. During flame spraying, the self-fluxing B and Si constituents spontaneously deoxidize and form slag, reducing oxide inclusions and enabling the molten particles to spread sufficiently and achieve metallurgical bonding with the substrate; the slag floats to the surface, ultimately yielding a dense, strongly bonded coating with wear and corrosion resistance. Figure 1b shows that the NiCr-Cr_3_C_2_ powder consists of spherical agglomerated-sintered composite particles composed of Cr_3_C_2_ hard phases and an NiCr binder phase, exhibiting good sphericity and uniform powder feeding [29]. During spraying, NiCr melts preferentially and encapsulates Cr_3_C_2_, resulting in a coating in which hard particles are embedded in a tough matrix. Figure 1c shows that the Ni-MoS_2_ powder exhibits a nearly spherical or irregular core–shell structure, with an Ni shell encapsulating the MoS_2_ core.

**Figure 2:**
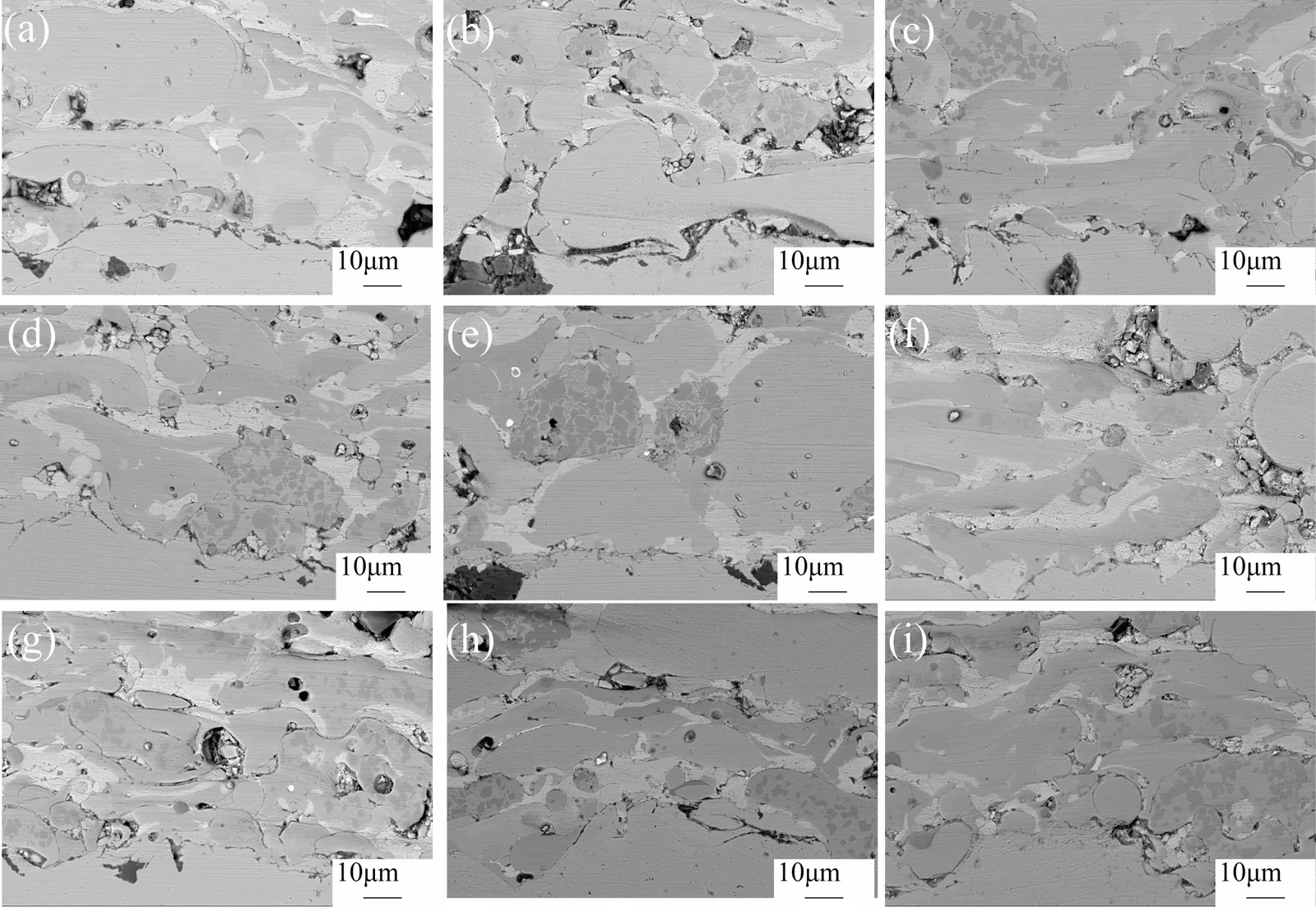
Cross-sectional SEM micrographs of the mixed coatings with different compositions: (**a**–**i**) correspond to C1 (70% Fe60 + 10% Ni-MoS_2_ + 20% NiCr-Cr_3_C_2_) to C9 (40% Fe60 + 20% Ni-MoS_2_ + 40% NiCr-Cr_3_C_2_).

Figure 2 shows the SEM micrographs of the mixed coatings with different proportions of Fe60, Ni-MoS_2_, and NiCr-Cr_3_C_2_. All coatings exhibit a pronounced lamellar or banded stacking structure, which is characteristic of the flame spraying process [30]. During spraying, molten or semi-molten powder particles impact the substrate surface at high velocity, flatten instantaneously into thin splats, and then rapidly solidify under the cooling effect of the substrate. Subsequent particles continuously impact the already-deposited coating surface and stack layer by layer, ultimately forming a lamellar structure parallel to the substrate interface. Between lamellae, fine interfacial boundaries often exist due to cooling shrinkage, oxide inclusions, or incompletely bonded pores, appearing as alternating bright and dark stripes in SEM backscattered mode. Although this lamellar structure may introduce anisotropy, it also effectively relieves residual stresses within the coating and prevents cracking of thick coatings [31].

The smooth regions in the images mainly originate from fully melted metallic binder phases. For instance, the Fe-Cr matrix in Fe60 and the NiCr alloy in NiCr-Cr_3_C_2_, owing to their relatively low melting points and good flowability, spread sufficiently after impacting the substrate and form dense, flat splats upon cooling, appearing as uniform bright gray bands under SEM [32]. In addition, the slag layer formed by the floating of slag generated by the self-fluxing B and Si constituents in Fe60 [33] also contributes to relatively smooth interfaces [34]. In contrast, the rough regions arise from unmelted or semi-molten hard particles that retain their angular shape and embed into the matrix upon impact, forming protrusions [35]; the MoS_2_ core in Ni-MoS_2_ particles decomposes or shrinks at high temperatures, forming microporous loose regions; oxides and silicate slag dispersedly distributed at lamellar boundaries increase roughness; and spray pores (from entrapped air or interparticle gaps) appear as black holes.

**Figure 3:**
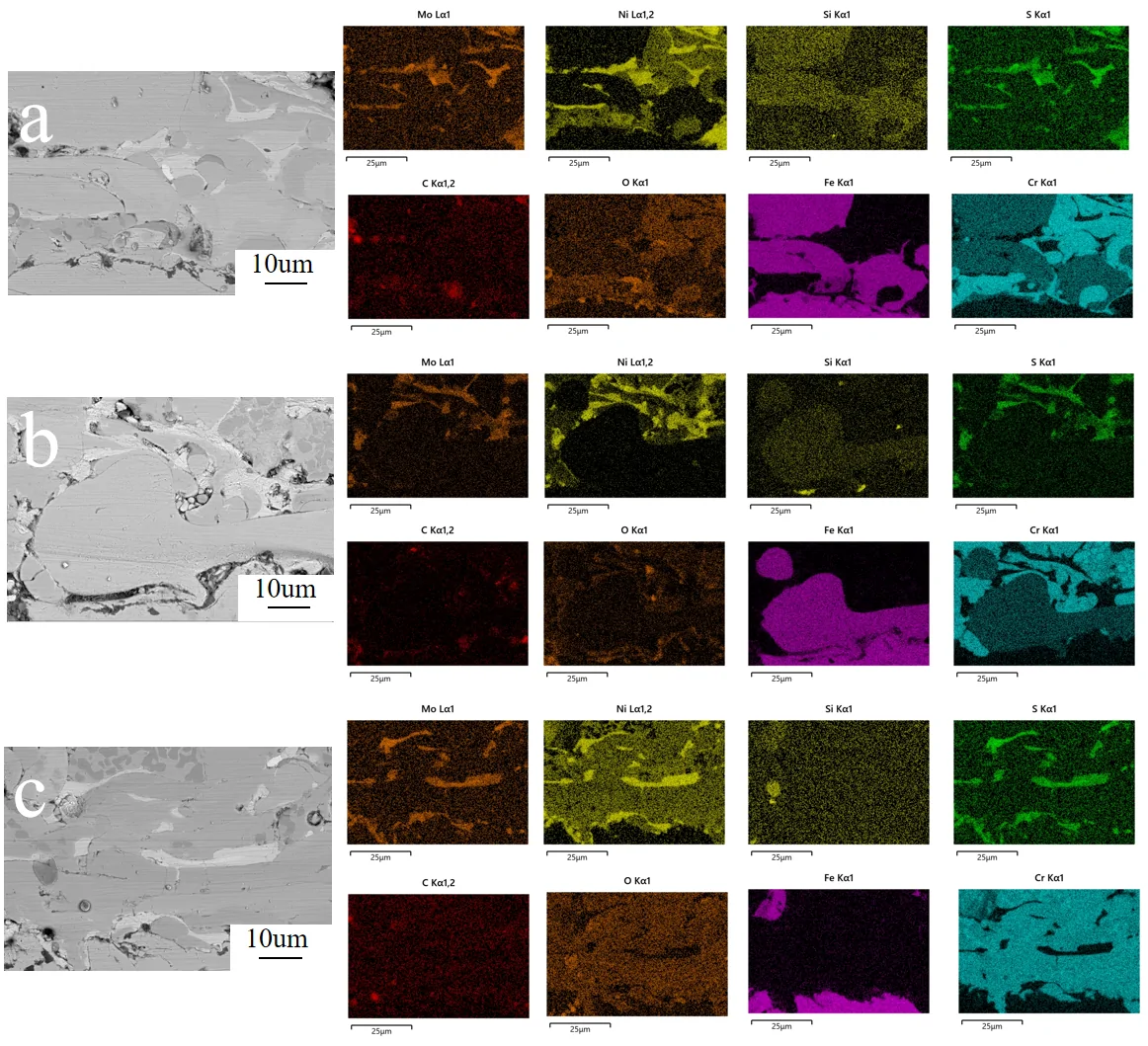
Cross-sectional SEM micrographs and EDS elemental mappings of the mixed coatings with different compositions: (**a**–**c**) correspond to C1 (70% Fe60 + 10% Ni-MoS_2_ + 20% NiCr-Cr_3_C_2_) to C3 (50% Fe60 + 10% Ni-MoS_2_ + 40% NiCr-Cr_3_C_2_).

**Figure 4:**
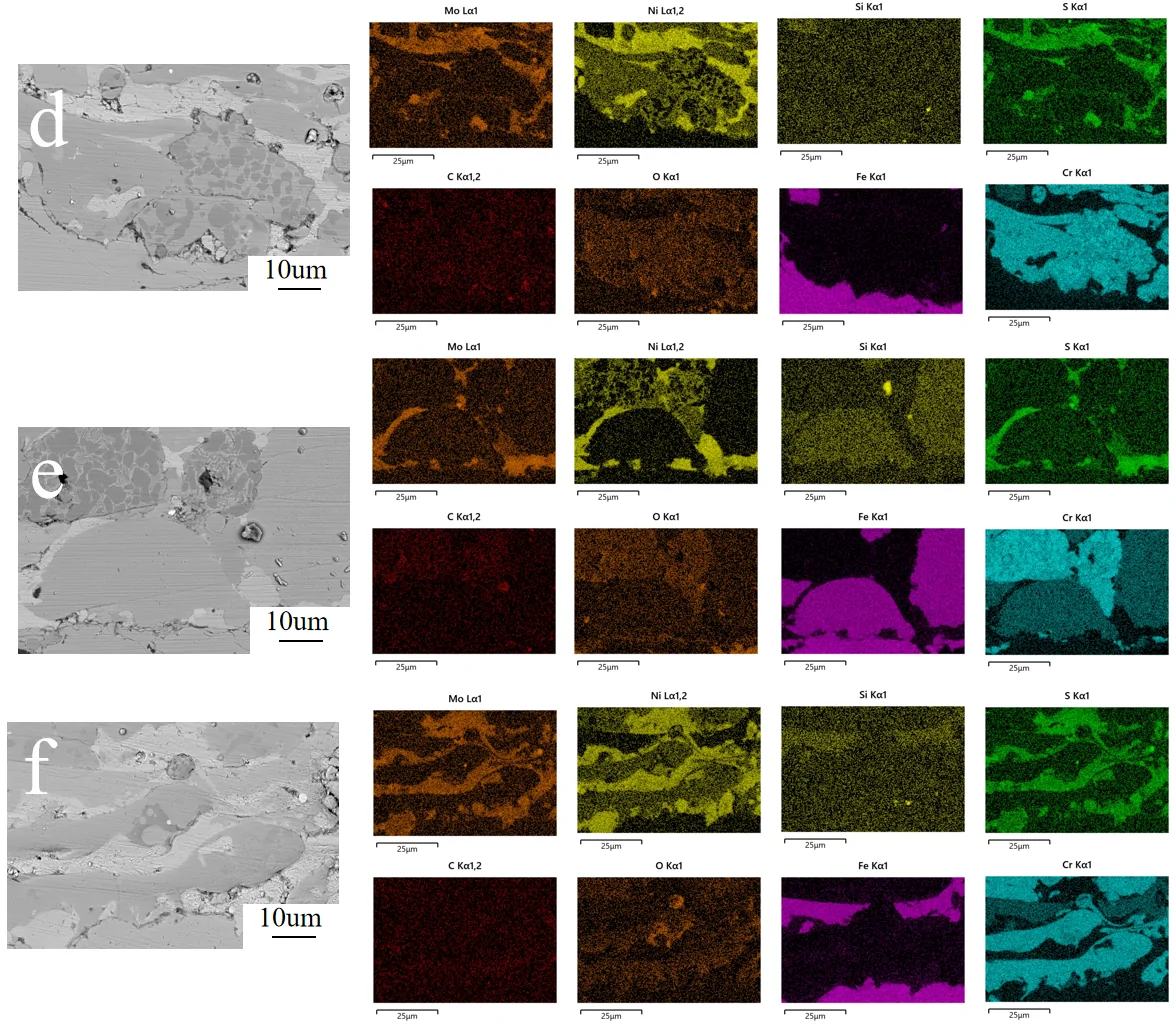
Cross-sectional SEM micrographs and EDS elemental mappings of the mixed coatings with different compositions: (**d**–**f**) correspond to C4 (65% Fe60 + 15% Ni-MoS_2_ + 20% NiCr-Cr_3_C_2_) to C6 (45% Fe60 + 15% Ni-MoS_2_ + 40% NiCr-Cr_3_C_2_).

**Figure 5:**
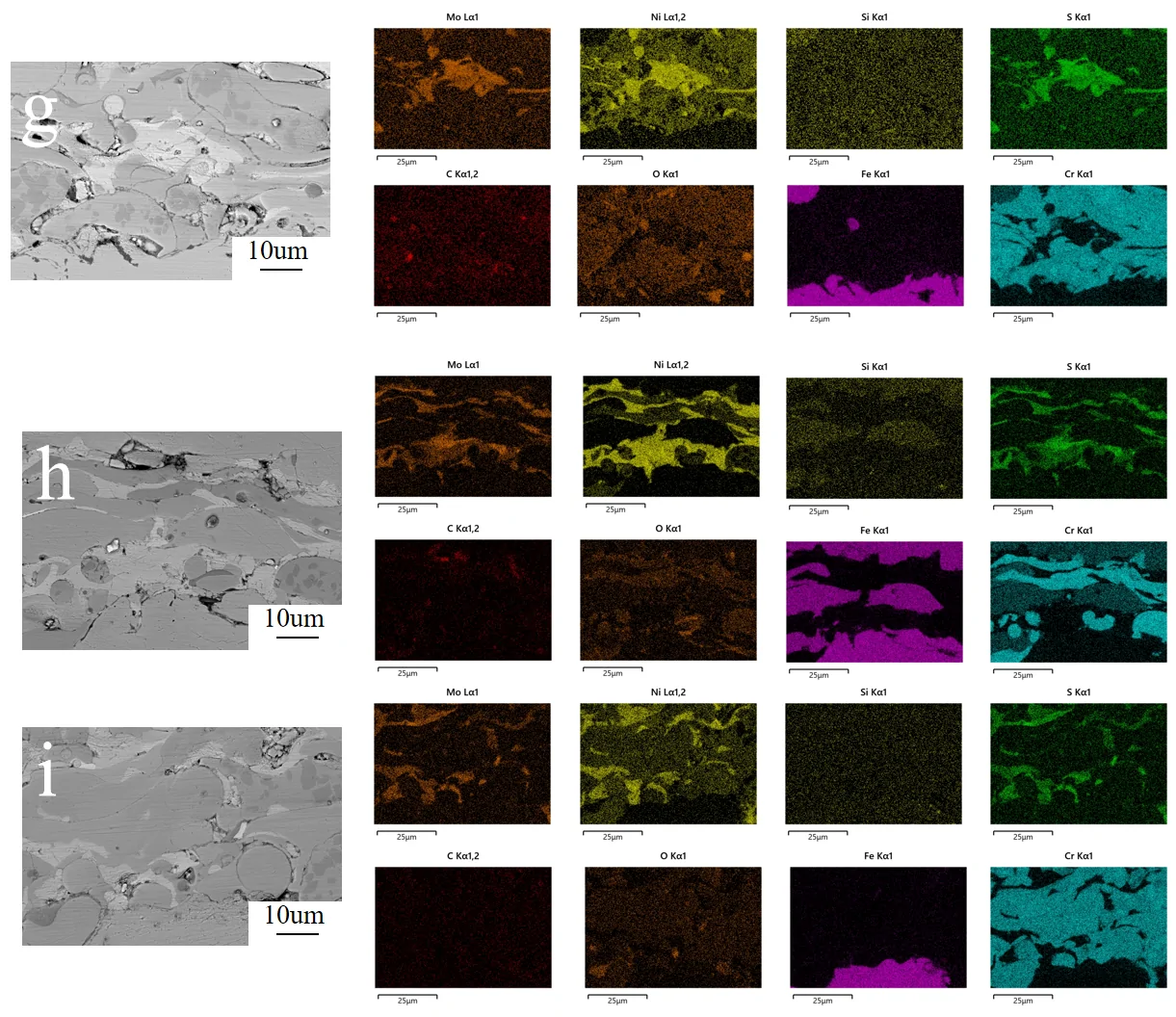
Cross-sectional SEM micrographs and EDS elemental mappings of the mixed coatings with different compositions: (**g**–**i**) correspond to C7 (60% Fe60 + 20% Ni-MoS_2_ + 20% NiCr-Cr_3_C_2_) to C9 (40% Fe60 + 20% Ni-MoS_2_ + 40% NiCr-Cr_3_C_2_).

Figure 3, Figure 4 and Figure 5 present the EDS elemental maps obtained by area scanning on randomly selected regions of the coatings with different compositions (C1–C9), which clearly reveal the evolution and synergistic interactions of the constituents during flame spraying: Fe is enriched as continuous bands, forming the load-bearing skeleton of the coating; Cr and Ni are highly overlapped in irregular blocky regions, confirming that the NiCr binder phase effectively encapsulates and agglomerates Cr_3_C_2_ hard particles to form dispersed hard-particle regions, while a portion of Cr dissolves in the Fe matrix to provide a strengthening effect; Mo and S exhibit consistent and dispersed distributions, indicating that the Ni shell effectively confines the Mo/S products within the coating to prevent excessive escape, while its metallic surface improves the wettability of the Mo/S-containing products with Fe60, promoting their uniform dispersion in the coating.

**Figure 6:**
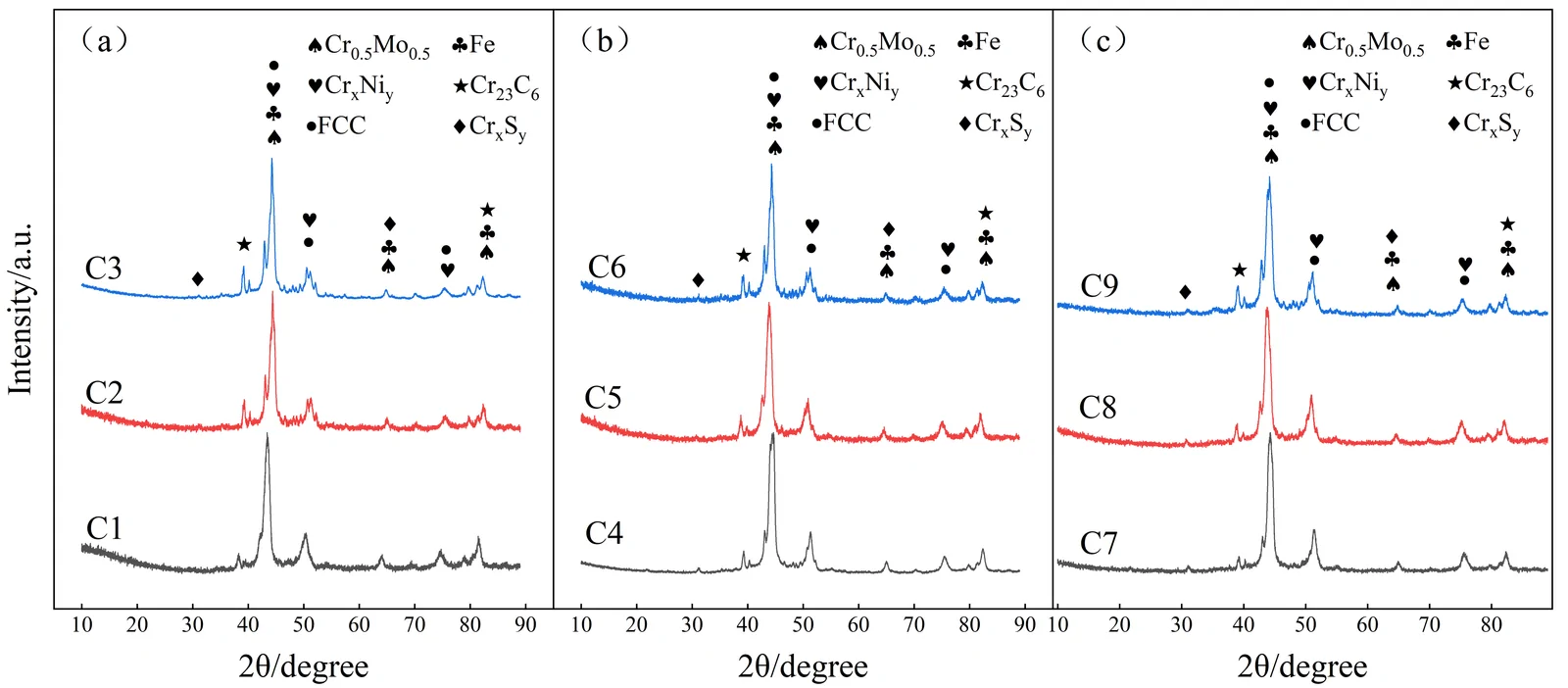
XRD patterns of the coatings with different compositions: (**a**) C1 (70% Fe60 + 10% Ni-MoS_2_ + 20% NiCr-Cr_3_C_2_) to C3 (50% Fe60 + 10% Ni-MoS_2_ + 40% NiCr-Cr_3_C_2_), (**b**) C4 (65% Fe60 + 15% Ni-MoS_2_ + 20% NiCr-Cr_3_C_2_) to C6 (45% Fe60 + 15% Ni-MoS_2_ + 40% NiCr-Cr_3_C_2_), and (**c**) C7 (60% Fe60 + 20% Ni-MoS_2_ + 20% NiCr-Cr_3_C_2_) to C9 (40% Fe60 + 20% Ni-MoS_2_ + 40% NiCr-Cr_3_C_2_).

The XRD phase analysis in Figure 6 reveals that the main phase in the coatings is Fe (JCPDS 06-0696), together with a Cr_0.5_Mo_0.5_ solid solution (PDF 65-9000), Cr*_x_*Ni*_γ_* (JCPDS 01-071-7594), Cr_23_C_6_ (JCPDS 35-0783), Cr*_x_*S*_γ_* (JCPDS 03-065-4651), and a small amount of a face-centered cubic (FCC) phase (JCPDS 47-1417) [36]. Notably, although an Ni shell was used in the raw powder to protect MoS_2_, no characteristic diffraction peaks of MoS_2_ were observed in the XRD patterns. Combined with the discussion in the Introduction, this result suggests that MoS_2_ underwent substantial thermal transformation and reactive conversion during spraying, resulting in the formation of Cr_0.5_Mo_0.5_ and Cr*_x_*S*_γ_* phases [37]. Furthermore, EDS elemental mapping of the sprayed coating cross-section (Figure 3) reveals the presence and relatively dispersed distribution of Mo and S, indicating that Mo/S-containing products were retained within the coating after spraying. These Mo/S-containing products may participate in the tribological process and contribute to reducing interfacial shear resistance, which is consistent with the observed variation in friction coefficient. Overall, the XRD and EDS results complement each other and provide evidence for the thermal transformation and distribution of MoS_2_-derived components in the coating, as well as their possible contribution to the friction-reduction behavior.

Table 7 summarizes the average microhardness values of the 65Mn substrate and the C1–C9 coatings. All composite coatings exhibited higher average microhardness than the 65Mn substrate, and C6 showed the highest average measured microhardness among the investigated coatings.

### 3.2. Tribological Performance Analysis

**Figure 7:**
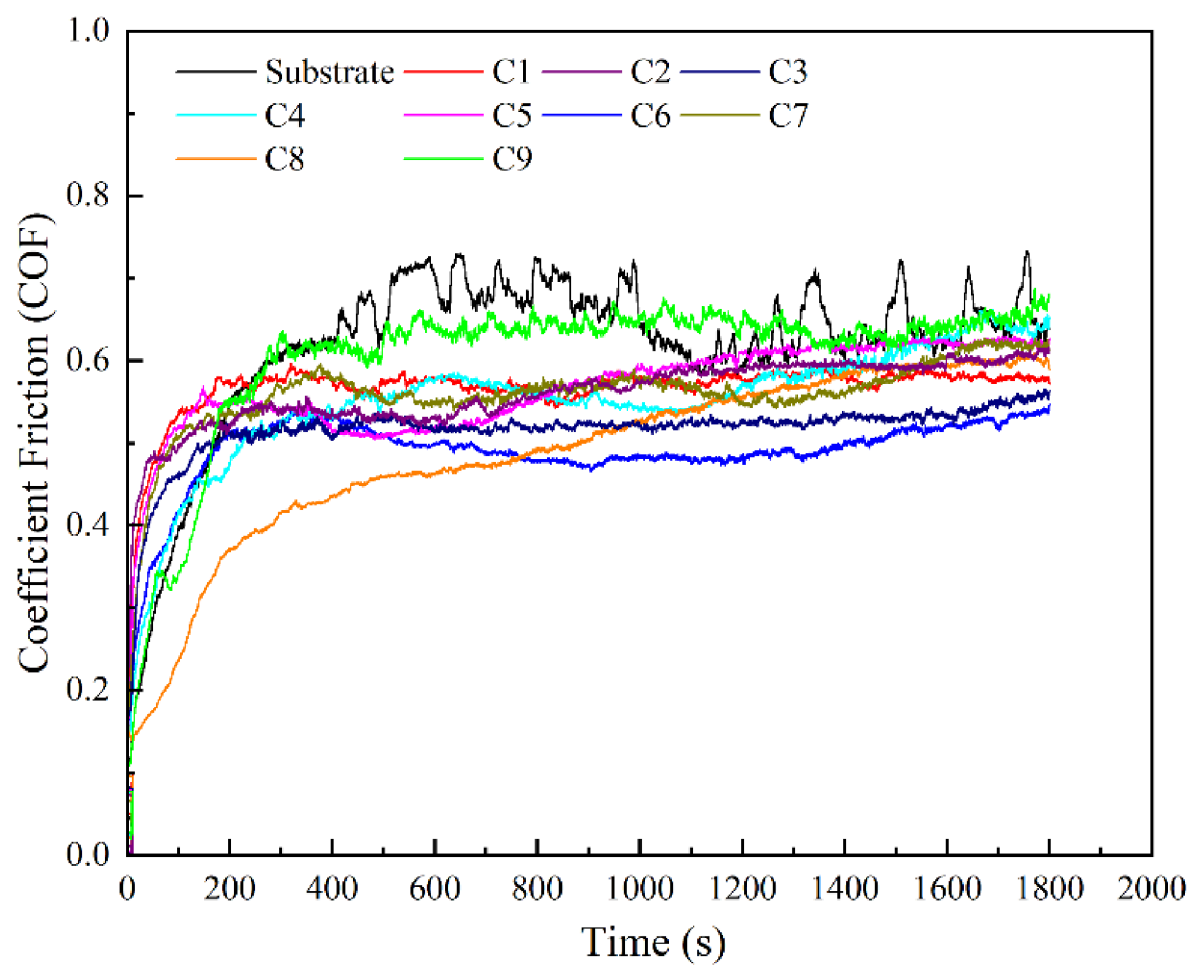
Coefficient of friction (COF) curves of the coatings with different compositions as a function of sliding time: C1 (70% Fe60 + 10% Ni-MoS_2_ + 20% NiCr-Cr_3_C_2_) to C9 (40% Fe60 + 20% Ni-MoS_2_ + 40% NiCr-Cr_3_C_2_).

Figure 7 shows the evolution of the friction coefficient with sliding time for the coatings with different compositions under a normal load of 40 N. At the initial stage of sliding, the rough coating surfaces and small real contact areas lead to high local contact stresses, causing the friction coefficient to rise rapidly; as sliding proceeds, the contact area gradually increases and the contact stress stabilizes, and the friction coefficients of all coatings enter a relatively steady stage after approximately 300 s.

**Figure 8:**
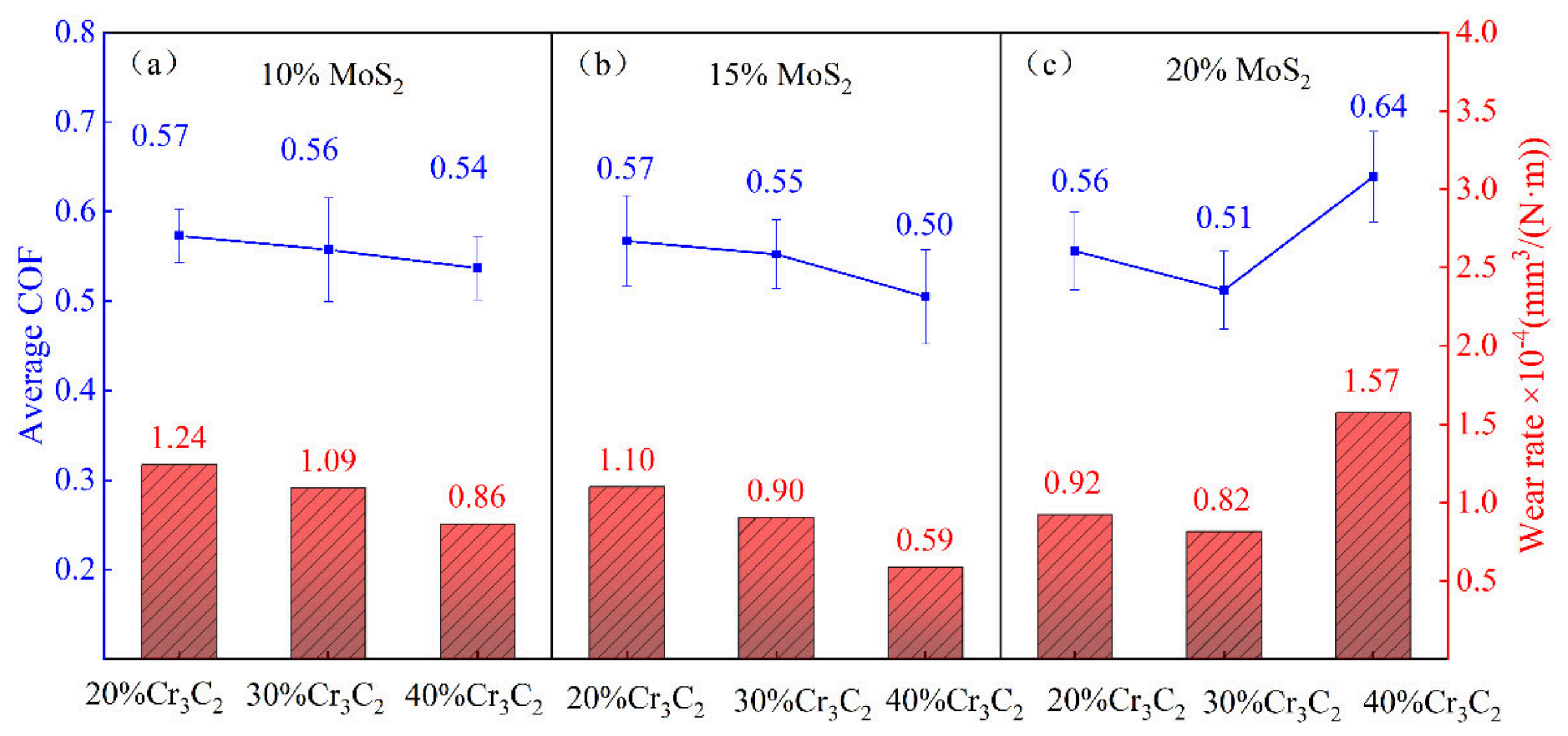
Average friction coefficient and wear rate of the coatings as a function of NiCr-Cr_3_C_2_ content: (**a**–**c**) correspond to the variation in NiCr-Cr_3_C_2_ content (20%, 30%, and 40%) when the Ni-MoS_2_ content is 10%, 15%, and 20%, respectively.

**Figure 9:**
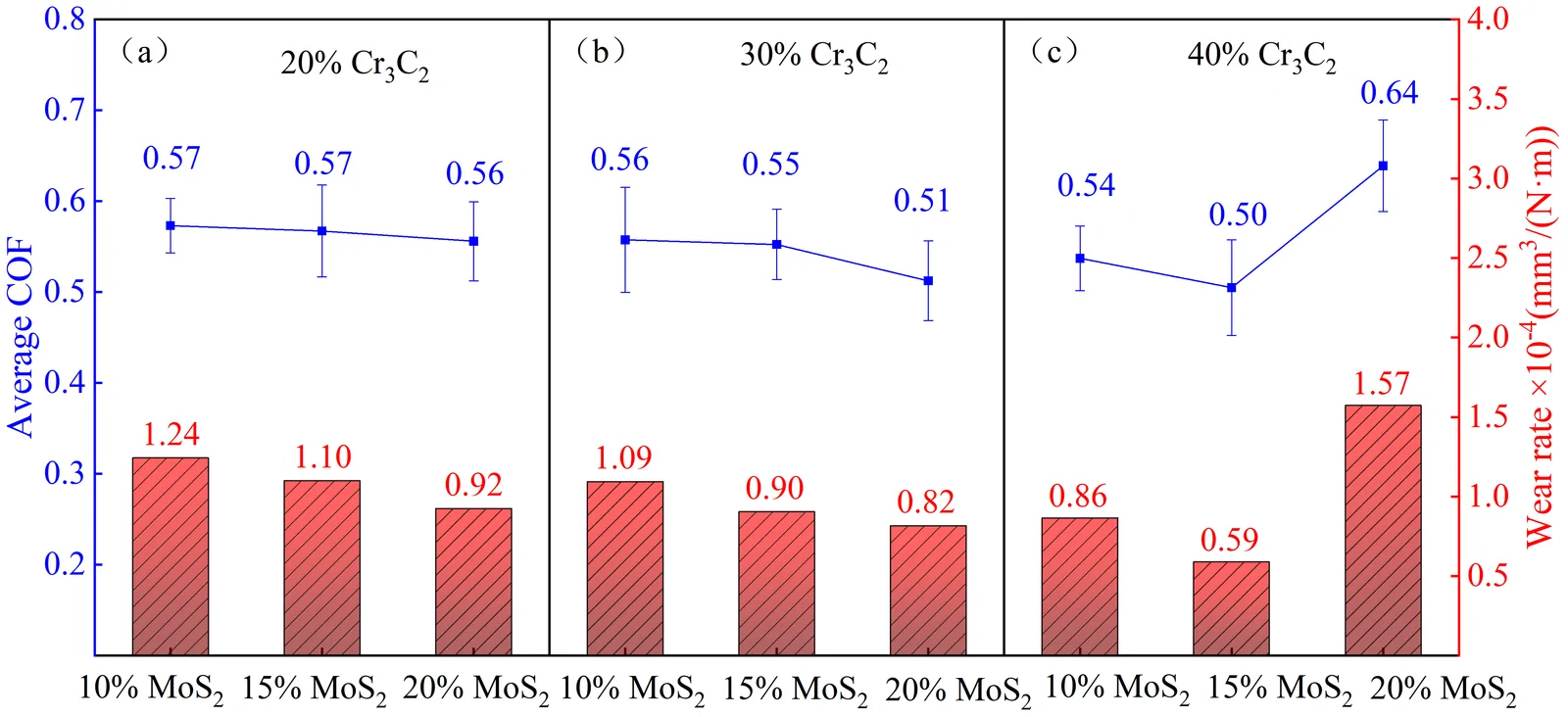
Average friction coefficient and wear rate of the coatings as a function of Ni-MoS_2_ content: (**a**–**c**) correspond to the variation in Ni-MoS_2_ content (10%, 15%, and 20%) when the NiCr-Cr_3_C_2_ content is 20%, 30%, and 40%, respectively.

Figure 8 and Figure 9 illustrate the effects of the relative Ni-MoS_2_ and NiCr-Cr_3_C_2_ contents on the average friction coefficient and wear rate. At Ni-MoS_2_ contents of 10 and 15 wt.%, increasing the NiCr-Cr_3_C_2_ content from 20 to 40 wt.% was accompanied by progressive reductions in both friction coefficient and wear rate, indicating the beneficial contribution of the Cr_3_C_2_-containing hard phase to load-bearing and wear resistance. At 20 wt.% Ni-MoS_2_, however, the improvement observed when the NiCr-Cr_3_C_2_ content increased from 20 to 30 wt.% was not maintained at 40 wt.%, as demonstrated by C9. Similarly, at 40 wt.% NiCr-Cr_3_C_2_, increasing the Ni-MoS_2_ content from 15 to 20 wt.% resulted in a pronounced increase in both friction coefficient and wear rate. These results indicate that the tribological response is governed by the combined proportions of the three constituents rather than by a simple monotonic effect of either additive alone. In C9, the simultaneous high contents of Ni-MoS_2_ and NiCr-Cr_3_C_2_ reduce the Fe60 matrix fraction to 40 wt.%, which may weaken matrix support and hard-particle retention and thereby promote local particle detachment and third-body abrasive action [38,39].

**Figure 10:**
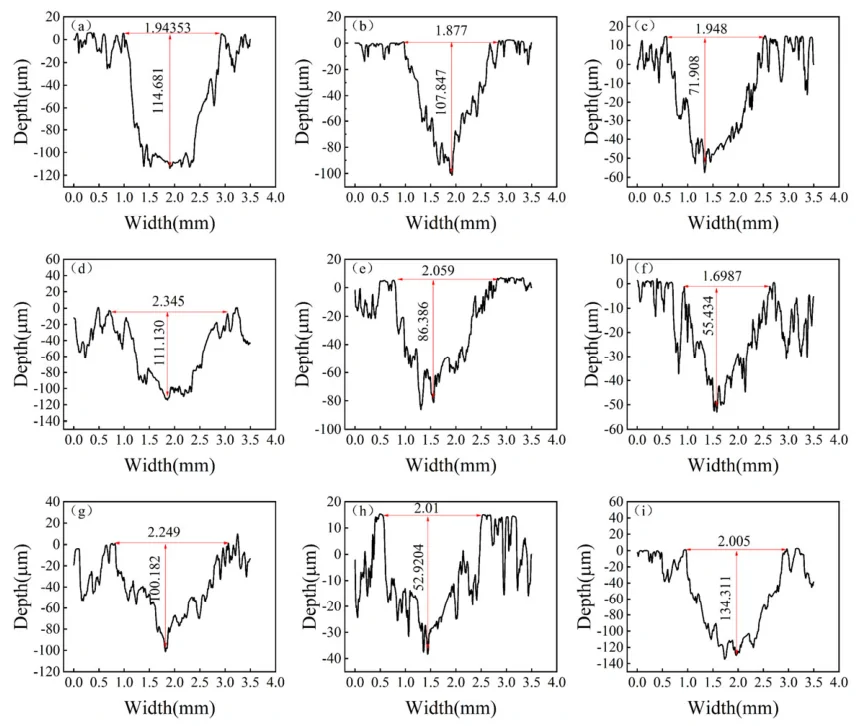
Wear track profiles of the coatings with different compositions: (**a**–**i**) correspond to C1 (70% Fe60 + 10% Ni-MoS_2_ + 20% NiCr-Cr_3_C_2_) to C9 (40% Fe60 + 20% Ni-MoS_2_ + 40% NiCr-Cr_3_C_2_).

To quantitatively correlate the wear-track geometry with the wear-rate trends shown in Figure 8 and Figure 9, two-dimensional cross-sectional profiles of the C1–C9 wear tracks were measured using a profilometer, as shown in Figure 10. C6 exhibited a relatively narrow and shallow wear track, with a width of 1.6987 mm and a maximum depth of 55.434 μm, corresponding to the lowest measured wear rate of 0.59 × 10^−4^ mm^3^/(N·m). In contrast, C9 exhibited a maximum wear depth of 134.311 μm and a width of 2.005 mm, corresponding to the highest wear rate of 1.57 × 10^−4^ mm^3^/(N·m). In addition, as the NiCr-Cr_3_C_2_ content increased from 20 to 40 wt.%, the maximum wear depth decreased from 114.681 to 71.908 μm for C1–C3 and from 111.130 to 55.434 μm for C4–C6. These profilometric results are consistent with the wear-rate variations shown in Figure 8 and Figure 9 and provide a quantitative geometrical comparison of wear severity among the coatings.

**Figure 11:**
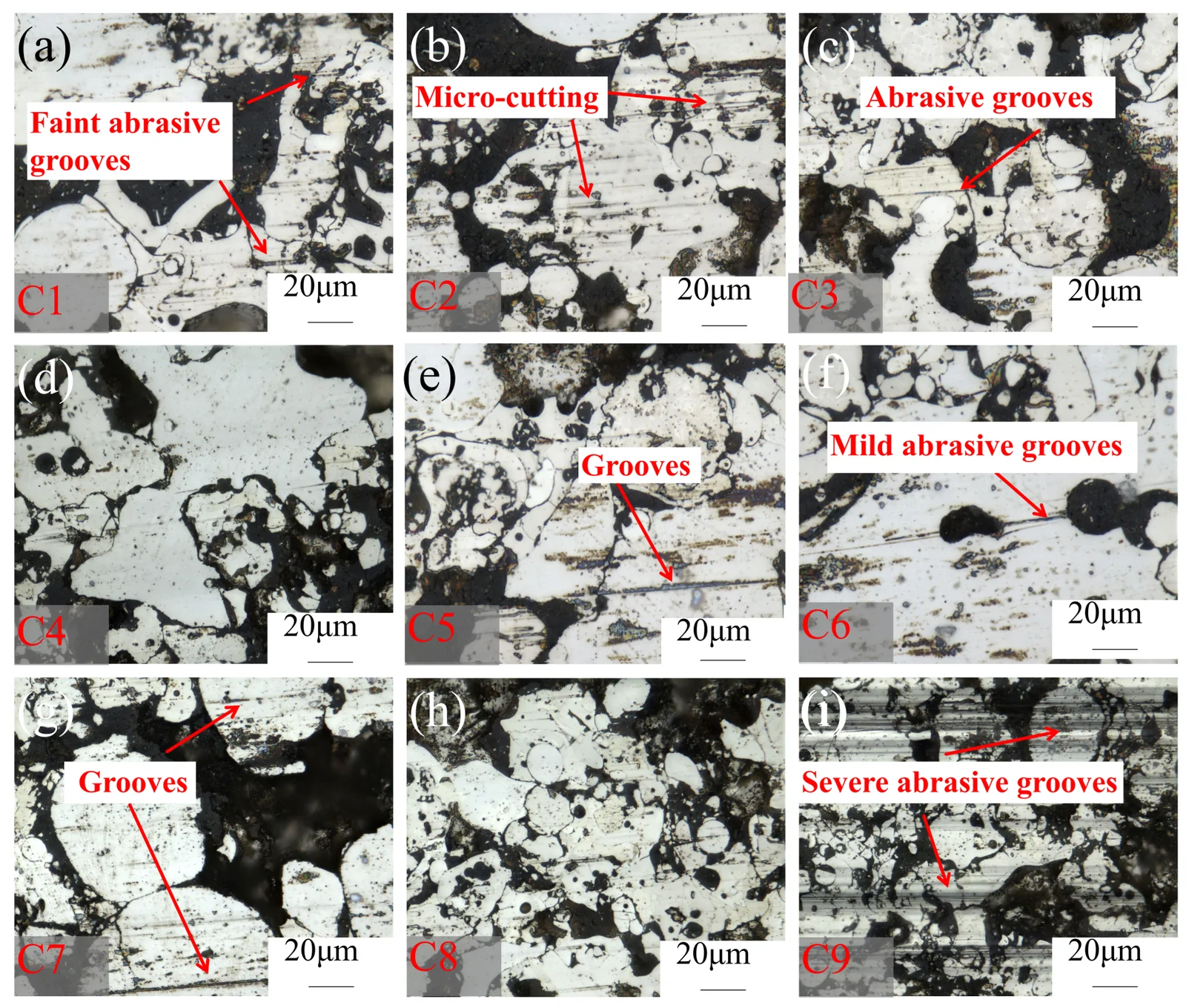
Wear track morphologies of the coatings with different compositions: (**a**–**i**) correspond to C1 (70% Fe60 + 10% Ni-MoS_2_ + 20% NiCr-Cr_3_C_2_) to C9 (40% Fe60 + 20% Ni-MoS_2_ + 40% NiCr-Cr_3_C_2_).

Figure 11 presents the worn-surface morphologies of the coatings with different compositions. The worn surfaces exhibit composition-dependent abrasive features, as indicated by the arrows in the figure. Faint abrasive grooves are observed on C1, while C2 shows distinct parallel scratches characteristic of micro-cutting. Abrasive grooves are also visible on C3, C5, and C7. In comparison, C6 exhibits relatively mild abrasive grooves, whereas C9 is characterized by pronounced, dense, and continuous abrasive grooves. These grooves are predominantly oriented along the sliding direction and provide direct morphological evidence that abrasive wear, involving grooving and micro-cutting, is the principal wear mechanism under the present dry reciprocating sliding conditions. In contrast, no sufficiently clear morphological features characteristic of dominant adhesive or oxidative wear were identified from the present worn-surface observations.

The severity of abrasive wear varies with coating composition. During sliding, the relatively softer Fe60 matrix may be preferentially removed, while the Cr_3_C_2_-containing hard phase provides load-bearing support and resistance to abrasive cutting. This effect is particularly evident for C6, which shows relatively mild abrasive damage and exhibits a low wear rate of 0.59 × 10^−4^ mm^3^/(N·m). In contrast, C9 displays much more pronounced abrasive grooves together with the highest measured wear rate of 1.57 × 10^−4^ mm^3^/(N·m). Because the Fe60 matrix fraction in C9 decreases to 40 wt.%, the mechanical support and constraint of the hard particles may be weakened. Local particle detachment could therefore occur more readily, and the detached hard particles may subsequently act as third-body abrasives, further intensifying micro-cutting and material removal.

Among the investigated compositions, C6 exhibits the lowest measured friction coefficient of 0.50 and wear rate of 0.59 × 10^−4^ mm^3^/(N·m). Its relatively mild abrasive morphology suggests that the combination of 40 wt.% NiCr-Cr_3_C_2_ and 15 wt.% Ni-MoS_2_ provides a favorable balance between load-bearing resistance and friction reduction. The NiCr-Cr_3_C_2_ phase contributes to resistance against abrasive cutting, whereas Mo/S-containing products derived from the Ni-MoS_2_ addition may contribute to reducing interfacial shear resistance. In contrast, the relatively high Ni-MoS_2_ content together with the reduced Fe60 matrix fraction in C9 may weaken the structural support of the coating, consistent with the severe abrasive features and increased wear observed for this composition.

The rule-of-mixtures concept provides a qualitative framework for understanding the balance among the matrix, hard reinforcing phase, and friction-reducing component. For a generic composite property, the overall response may be schematically expressed as (*P*_c_ ≈ *V*_m_*P*_m_ *+ V*_h_*P*_h_ *+ V*_l_*P*_l_) where (*P*_c_) represents the overall property of the composite, (*P*_m_), (*P*_h_), and (*P*_l_) represent the corresponding property contributions of the matrix, hard phase, and friction-reducing component, respectively, and (*V*_m_), (*V*_h_), and (*V*_l_) denote their corresponding volume fractions. In the present study, this expression is used only as a qualitative conceptual framework rather than as a quantitative predictive model, because the tribological response is additionally affected by phase distribution, interfacial bonding, microstructural integrity, and phase transformation during spraying and sliding.

In the present coating system, the NiCr-Cr_3_C_2_ phase acts as a load-bearing hard constituent and contributes to resistance against plastic deformation and abrasive wear, whereas Mo/S-containing products derived from the Ni-MoS_2_ addition may contribute to reducing interfacial shear resistance [40,41]. Therefore, increasing the hard-phase content generally favors load-bearing and wear resistance, while an appropriate amount of the Ni-MoS_2_-derived component may contribute to friction reduction. However, excessive changes in the relative proportions of these constituents may disturb the balance between mechanical support and friction reduction. Accordingly, the actual tribological behavior cannot be described by a simple linear rule of mixtures. Among the investigated compositions, C6 containing 15 wt.% Ni-MoS_2_ and 40 wt.% NiCr-Cr_3_C_2_ exhibited a favorable balance among these contributions, with a friction coefficient of 0.50 and a wear rate of 0.59 × 10^−4^ mm^3^/(N·m).

**Figure 12:**
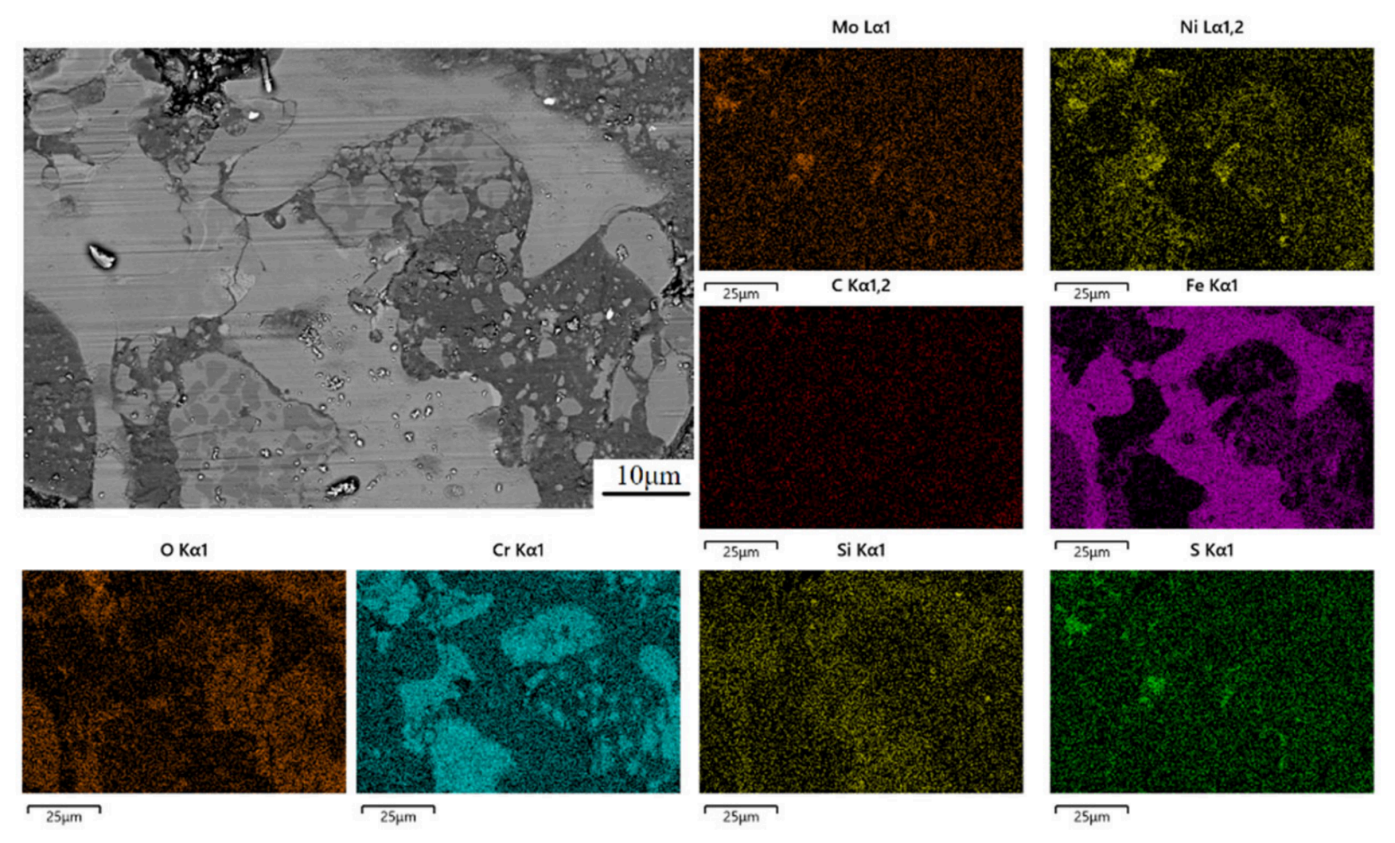
EDS elemental distribution of the worn surface of the C6 coating (45% Fe60 + 15% Ni-MoS_2_ + 40% NiCr-Cr_3_C_2_) after friction and wear testing.

To further elucidate the wear behavior of the C6 coating, Figure 12 presents the EDS elemental distribution on its worn surface. Cr- and Ni-enriched regions remain identifiable within the worn area, indicating the retention of Cr_3_C_2_/NiCr-containing hard-phase regions during sliding. Mo and S are also distributed over the worn surface and exhibit a similar spatial distribution, indicating the presence of Mo/S-containing products after wear. These Mo/S-containing products may contribute to reducing interfacial shear resistance, while the Cr/Ni-rich hard-phase regions provide load-bearing support and resistance to abrasive cutting. No obvious large-scale spalling or severe agglomeration is observed in the examined region, which is consistent with the relatively mild abrasive morphology and the low friction coefficient and wear rate of C6. In addition, C6 exhibited the highest average measured microhardness among the investigated coatings, which is consistent with its relatively strong resistance to plastic deformation and abrasive material removal.

## 4. Discussion

### 4.1. Effect of Composition on Hardness and Tribological Behavior

The tribological response of the Fe60-based composite coatings was strongly dependent on the relative contents of NiCr-Cr_3_C_2_, Ni-MoS_2_, and the Fe60 matrix. At Ni-MoS_2_ contents of 10 and 15 wt.%, increasing the NiCr-Cr_3_C_2_ content generally improved the wear resistance of the coatings, which is consistent with the load-bearing contribution of the Cr_3_C_2_-containing hard phase. Cr_3_C_2_ provides resistance to plastic deformation, ploughing, and micro-cutting during sliding, while the NiCr binder phase and Fe60 matrix contribute to the mechanical support and retention of the hard particles. C6 exhibited the highest average measured microhardness among the investigated formulations. However, this beneficial trend did not continue for C9 at 20 wt.% Ni-MoS_2_, indicating that the overall tribological response depends on the balance among all three constituents rather than on the NiCr-Cr_3_C_2_ content alone. This effect can be attributed primarily to the high hardness of the Cr_3_C_2_ phase, which provides resistance to plastic deformation, ploughing, and micro-cutting during sliding. The NiCr binder phase and Fe60 matrix further contribute to the mechanical support and retention of the hard particles. Consistent with this interpretation, the C6 coating exhibited the highest measured microhardness among the investigated formulations.

The effect of Ni-MoS_2_ was non-monotonic. At moderate addition levels, increasing the Ni-MoS_2_ content was accompanied by a reduction in friction coefficient, indicating that Mo/S-containing components derived from the Ni-MoS_2_ addition may contribute to reducing interfacial shear resistance during sliding. However, when the Ni-MoS_2_ content reached 20 wt.%, the Fe60 matrix was further reduced, and the resulting decrease in the relative matrix fraction may have weakened the mechanical support of the composite structure. This effect was particularly evident for C9, which contained 20 wt.% Ni-MoS_2_ and 40 wt.% NiCr-Cr_3_C_2_. Its wear-track profile showed the greatest maximum wear depth (134.311 μm) and a relatively large width (2.005 mm), together with the highest measured wear rate of 1.57 × 10^−4^ mm^3^/(N·m). The deep and long grooves observed in the wear track further indicate intensified third-body abrasive action, which may be associated with local detachment of hard particles and subsequent abrasive cutting.

### 4.2. Relationship Between Microstructure, Phase Evolution, and Wear Behavior

The wear-track profiles and worn-surface characterization provide further evidence for the different roles of the constituent phases. Increasing the NiCr-Cr_3_C_2_ content from 20 to 40 wt.% reduced the maximum wear depth from 114.681 to 71.908 μm for C1–C3 and from 111.130 to 55.434 μm for C4–C6. The relatively shallow and narrow wear track of C6, together with its wear rate of 0.59 × 10^−4^ mm^3^/(N·m), indicates that a sufficiently high fraction of the hard phase can effectively reduce severe surface deformation and material removal. In contrast, the substantially deeper wear track of C9 indicates that excessive reduction of the Fe60 matrix, combined with a high Ni-MoS_2_ content, may compromise the structural integrity of the coating. These observations are consistent with the SEM morphology, where grooves parallel to the sliding direction are evident and abrasive wear appears to be an important wear mechanism.

The XRD and EDS results provide complementary information concerning the evolution of the MoS_2_-derived components. No characteristic crystalline MoS_2_ diffraction peaks were detected, whereas Cr-Mo- and Cr-S-containing phases were identified. Together with the Mo and S distributions observed by EDS, these results suggest that the Ni-MoS_2_ component underwent substantial thermal transformation and reactive conversion during flame spraying, with Mo/S-containing products retained in the coating and subsequently present in the worn region. These products may contribute to the observed friction-reduction behavior during sliding. Meanwhile, the Cr_3_C_2_-containing hard phase remained associated with Cr/Ni-enriched regions and provided load-bearing support within the Fe60 matrix. Thus, the observed friction and wear behavior is better understood as the combined result of the hard-phase load-bearing effect, the contribution of Mo/S-containing products to friction reduction, and the structural support provided by the Fe60 matrix, rather than as the consequence of a single phase or mechanism.

### 4.3. Performance of the C6 Formulation and Limitations of the Present Study

Among the nine investigated compositions, C6 (45 wt.% Fe60 + 15 wt.% Ni-MoS_2_ + 40 wt.% NiCr-Cr_3_C_2_) exhibited the lowest measured friction coefficient (0.50) and wear rate (0.59 × 10^−4^ mm^3^/(N·m)), together with the highest measured microhardness. Therefore, C6 can be regarded as the best-performing formulation within the investigated composition range and under the present test conditions. This result suggests that the combination of a relatively high NiCr-Cr_3_C_2_ content and a moderate Ni-MoS_2_ content can provide a favorable balance between load-bearing capacity and friction reduction. However, the present experiments were conducted under a specific dry reciprocating sliding condition (40 N normal load, 5 Hz, 6 mm stroke, and 30 min at room temperature). In addition, the nine compositions were evaluated within discrete composition levels rather than through a statistically optimized continuous composition space. Therefore, the C6 composition should not be interpreted as a universal optimum for all service conditions. Further investigations involving repeated measurements with statistical significance analysis, longer-duration sliding tests, different loads and sliding speeds, and elevated-temperature wear conditions are required to evaluate the robustness and broader applicability of the composition-property relationship.

## 5. Conclusions

(1) Flame spraying successfully produced Fe60-based composite coatings containing NiCr-Cr_3_C_2_ and Ni-MoS_2_ on the 65Mn substrate. SEM and EDS analyses showed a typical layered thermal-sprayed morphology, with the constituent elements distributed within the coatings. All composite coatings exhibited higher average microhardness than the 65Mn substrate. The Cr_3_C_2_-containing phase contributed to the load-bearing and abrasive-wear resistance of the coatings, whereas the introduction of Ni-MoS_2_-derived Mo/S-containing components was associated with the observed friction-reduction behavior.

(2) XRD analysis revealed Fe as the main phase together with Cr-Mo-, Cr-Ni-, Cr-C-, and Cr-S-containing phases, while no characteristic crystalline MoS_2_ peaks were detected. Combined with the EDS results, these observations suggest that Ni-MoS_2_ underwent substantial thermal transformation and reactive conversion during spraying. The resulting Mo/S-containing products may contribute to the friction-reduction behavior during subsequent sliding.

(3) The friction coefficient and wear rate exhibited a non-monotonic dependence on composition. Increasing the NiCr-Cr_3_C_2_ content generally reduced wear severity by increasing the load-bearing capability of the coating, whereas excessive addition of Ni-MoS_2_ and simultaneous reduction of the Fe60 matrix could weaken the structural support of the composite and promote local particle detachment and third-body abrasive action. The two-dimensional wear-track profiles were consistent with the measured wear rates, with C6 showing a relatively shallow wear track and C9 exhibiting the most severe wear.

(4) Among the nine investigated formulations, C6 (45 wt.% Fe60 + 15 wt.% Ni-MoS_2_ + 40 wt.% NiCr-Cr_3_C_2_) showed the best measured tribological performance under the present experimental conditions, with a friction coefficient of 0.50 and a wear rate of 0.59 × 10^−4^ mm^3^/(N·m). The result indicates that an appropriate balance between the hard NiCr-Cr_3_C_2_ phase, Mo/S-containing components, and Fe60 matrix is important for simultaneously reducing friction and wear. However, the present conclusion is limited to the investigated composition range and room-temperature dry reciprocating conditions. Long-duration, elevated-temperature, and variable-load wear tests, together with repeated measurements and statistical analysis, are still required to further establish the stability and general applicability of the observed composition–property relationship.

## Figures and Tables

**Table 1:** Chemical composition of Fe60 powder.

Element	C	Si	B	Cr	Ni	Fe
mass fraction/%	0.8–1.2	1.0–2.0	3.8–4.2	16–18	9.0–12	balance

**Table 2:** Chemical composition of NiCr-Cr_3_C_2_ powder.

Element	Cr	Ni	C	Fe	O
mass fraction/%	68.84	21.36	9.69	0.06	0.05

**Table 3:** Chemical composition of Ni-MoS_2_ powder.

Element	MoS_2_	Ni
mass fraction/%	balance	72–80

**Table 4:** Composition of the coating specimens.

Sample	Mass Fraction/%								
	C1	C2	C3	C4	C5	C6	C7	C8	C9
Fe60	70	60	50	65	55	45	60	50	40
Ni-MoS_2_	10	10	10	15	15	15	20	20	20
NiCr-Cr_3_C_2_	20	30	40	20	30	40	20	30	40

**Table 5:** Chemical composition of the 65Mn substrate.

Element	C	Si	Mn	Fe
mass fraction/%	0.62–0.70	0.17–0.37	0.90–1.20	balance

**Table 6:** Flame spraying parameters.

Parameter	Propane Flow Rate (L/min)	Oxygen Flow Rate (L/min)	Traverse Speed (mm/s)	Powder Feed Rate (g/min)	Spray Distance (mm)
Value	19	12	350	50	130

**Table 7:** Average microhardness of the 65Mn substrate and C1–C9 coatings.

Specimen	Average Microhardness (HV_0.5_)
65Mn substrate	388.1
C1	660.9
C2	719.1
C3	704.4
C4	607.8
C5	630.1
C6	724.4
C7	660.3
C8	642.4
C9	682.3

## Data Availability

The original contributions presented in this study are included in the article. Further inquiries can be directed to the corresponding author.
